# The moderating roles of bedtime activities and anxiety/depression in the relationship between attention-deficit/hyperactivity disorder symptoms and sleep problems in children

**DOI:** 10.1186/s12888-018-1879-4

**Published:** 2018-09-17

**Authors:** Lian Tong, Yan Ye, Qiong Yan

**Affiliations:** 1 Department of Maternal, China and Adolescent Health, School of Public Health, Fudan University/Key laboratory Public Health Safety, Chinese Ministry of Education, P.O. Box 244, 138 Yixueyuan Road, Shanghai, 200032 China; 2 Department of Behavior and Psychology Science, Zhejiang University, Hangzhou, China

**Keywords:** ADHD, Sleep problems, Bedtime activities, Anxiety/depression

## Abstract

**Background:**

Children with attention-deficit/hyperactivity disorder (ADHD) often experience sleep problems, but the comorbidity mechanism has not been sufficiently studied. This study aimed to determine the comorbidity of ADHD symptoms and sleep problems as well as the moderating effects of bedtime activities and depression/anxiety symptoms on the relationship between ADHD symptoms and sleep problems.

**Methods:**

We recruited 934 primary students from third to fifth grade and their parents by stratified random sampling from three primary schools in Shanghai, China. This study used parent-reported versions of the ADHD Rating Scale-IV, Children’s Sleep Habits Questionnaire, and Achenbach Child Behavior Checklist. We used hierarchical linear regression analysis to clarify the moderating effects of bedtime activities and depression/anxiety symptoms.

**Results:**

We found that children with more ADHD symptoms had shorter sleep durations and more sleep problems on weekdays. Screen time before bedtime strengthened the relationship between ADHD symptoms and sleep-disordered breathing. Children with more screen time were more likely to have sleep onset delay, while those with less screen time had more sleep onset problems with increasing ADHD symptoms. The high bedtime eating group experienced more night waking with increasing ADHD symptoms compared with the low bedtime eating group. Anxiety/depression exacerbated total sleep problems and further interacted with ADHD symptoms to predict sleep length and sleep duration problems.

**Conclusions:**

Bedtime activities and emotional problems had important moderating effects on the relationship between ADHD symptoms and sleep problems. These findings indicate that appropriate bedtime management and emotional management may reduce sleep problems and improve sleep duration for children with ADHD symptoms.

## Background

Attention-deficit/hyperactivity disorder (ADHD) is a common neurodevelopmental disorder among children, characterized by hyperactivity, inattention, and impulsivity. It affects 5–10% of school-age children worldwide [1–3]. ADHD is commonly associated with a number of other behavioral and emotional disorders, such as conduct disorder, anxiety disorder, depression disorder [4, 5], and sleep problems, one of the most common comorbidities [6]. Children with ADHD symptoms are more likely to report sleep problems such as prolonged sleep onset latency, difficulty falling asleep, bedtime resistance problems, sleep-disordered breathing, restless leg movements during sleep, night awakenings, and difficulties with morning awakening [7, 8]. As many as 55–74% of children with ADHD are affected by sleep disturbances [9]. A population-based study with a sample of 9486 adolescents found that ADHD symptoms were linked to shorter sleep duration, longer sleep latency, longer nocturnal wake time, and sleep deficiency [10].

The underlying mechanism of comorbid ADHD and sleep problems is complicated. A biopsychosocial and contextual model of sleep suggests that biological factors, psychosocial factors (i.e., mental health, family, and peer factors), and contextual factors (i.e., electronic media use, homework, and extracurricular activities) contribute to the association of ADHD symptoms and sleep problems [8]. The present study narrowly focuses on mental health problems of anxiety/depression and bedtime activities including screen time and diet behaviors. Evidence from epidemiological and clinical studies has suggested that lifestyle behaviors, such as screen time, diet, and physical activity, are closely correlated with both ADHD symptoms and sleep problems [11]. One study found that children with ADHD symptoms were almost twice as likely to have unhealthy behaviors compared with typically developing children. For example, children with ADHD symptoms spent more time watching TV, playing video games, and using computers and consumed more sugar and artificially sweetened juice than their counterparts [12]. A meta-analysis of 45 empirical studies has suggested that there is a significant relationship between ADHD symptoms and media use [13].

As is well known, for typical children and adolescents worldwide, exposure to the Internet, computers, video gaming, and caffeine is related to various sleep problems [14]. Longitudinal research also has shown a link between electronic media use and sleep problems [15]. The mechanisms beneath this phenomenon may be explained by a model [16]. According to this model, electronic media use may replace children’s sleep time, or may deteriorate sleep quality through increased psychophysiological arousal leading by the contents of the media or through too much light exposure. However, little is known about how increased screen time is related to sleep problems in children with ADHD. A recent study found that playing games significantly reduced the average sleep hours of boys with ADHD, although no such relationship was found among typically developing boys [17]. Another study found that the use of an electronic device for 1 hour before bedtime did not influence the relationship between ADHD symptoms and sleep problems in teenagers [10]. Thus, a clear conclusion cannot be drawn from the inconsistent findings of previous studies. There has been particularly little research on the effects of screen exposure before bedtime in children with ADHD.

In addition, increased screen time is associated with unhealthy eating habits, including lower vegetable consumption, higher consumption of unhealthy snacks and drinks, higher consumption of fast food, and higher overall caloric intake [18]. As screen activities such as watching TV, playing video games, and using mobile phones have become predominant bedtime activities for children [19], this has led to more night eating. Our recent study indicated that children with ADHD have more screen time, and more eating behaviors co-occur with screen time than children without ADHD symptoms [20]. A clinical study showed that treatment with an elimination diet reduced sleep complaints and physical problems in children diagnosed with ADHD [21]. This finding suggests that food consumption may be related to sleep problems in children with ADHD. However, it remains unknown whether bedtime eating contributes to sleep problems in the context of ADHD.

Beyond lifestyle-related behaviors, the combination of ADHD and emotional problems in children leads to more sleep problems. Studies have indicated that more than 50% of children with ADHD suffer from anxiety/depression problems that contribute to specific sleep problems [22, 23]. Youth with comorbid ADHD and anxiety were found to have the longest sleep onset delay, shortest sleep duration, and greatest daytime sleepiness in comparison to typically developing youth and youth with ADHD alone [24]. A clinical study found that children with ADHD comorbid with anxiety/depression showed more total sleep problems, difficulty falling asleep, restlessness during sleep, waking during the night, nightmares, walking or talking during sleep, waking too early, and sleeping less than typical children [25]. A large population-based study suggested that ADHD symptoms and anxiety/depression symptoms independently contribute to delayed sleep phase syndrome in adolescents [26]. However, it is still unknown whether ADHD and anxiety/depression contribute to sleep problems in children separately or via an interactive effect between these factors.

The first purpose of this study is to explore the relationship between screen time and eating behaviors before bedtime with sleep problems, particularly the interaction between ADHD and bedtime activities. The second purpose of this study is to clarify the moderating role of anxiety/depression in the relationship between ADHD symptoms and sleep problems.

## Methods

### Participants

We recruited 934 primary students, ages 9 to 12 years old (mean = 10.5, *SD* = 1.1), and their parents by stratified random sampling of three primary schools (third grade to fifth grade) in Shanghai, China. The sample covered different socioeconomic strata in China. Parents in School A had the highest socioeconomic status (SES; e.g., high educational attainment, high household income, and young parents), and parents in School C had the lowest SES. Of the students, 501 (53.8%) were boys and 430 (46.2%) were girls.

### Measures

Both a student self-report questionnaire and a parent-completed questionnaire were used in this study. The two questionnaires were matched by student identification (ID). The specific assessment instruments used in this study were as follows:

#### ADHD symptoms

ADHD symptoms were assessed by the parent-reported version of the ADHD Rating Scale-IV (ADHDRS-IV) [27]. The ADHDRS-IV is an 18-item ADHD assessment scale, and it consisted of two subscales, inattention and hyperactivity-impulsivity, each containing nine items. For example, the following items were used to assess hyperactivity and impulsivity symptoms: 1) Fidgets with hands or feet or squirms in seat, 2) Is “on the go” or acts as if “driven by a motor”, 3) Runs about or climbs excessively in situations in which it is inappropriate; The items, like “Fails to give close attention to details or makes careless mistakes in schoolwork”, “Loses things necessary for tasks or activities”, and “Is easily distracted” were used to evaluate inattention symptoms. Each item mapped onto one of the 18 DSM-IV (Diagnostic and Statistical Manual of Mental Disorders-IV) symptoms of ADHD. Parents were required to rate the frequency of each of the ADHD symptoms occurred over the past 6 months on a five-point Likert scale with 0 for never or rarely, 1 for sometimes, 2 for often, and 3 for very often. The sum of all the scores on the 18 items results in a total score. The reliability and validity of the home version of the ADHDRS-IV had been verified in a sample of Chinese children ages 6–17 years old [28]. Cronbach’ s alpha of the ADHDRS-IV for the present sample was 0.92. A cutoff of 26 points was used to define children with or without clinically significant ADHD symptoms [28].

#### Sleep problems and sleep length

Parents completed the Children’s Sleep Habits Questionnaire (CSHQ) to report their children’s sleep problems [29]. The CSHQ contains 45 (only 33 items for scoring) items in eight subscales. There are bedtime resistance (six items, e.g., goes to bed at same time), sleep onset delay (one item, falls asleep in 20 min), sleep duration (three items, e.g. sleep the right amount), sleep anxiety (four items, e.g. afraid of sleeping alone), night waking (three items, e.g. awakes more than once), parasomnias (seven items, e.g. talks during sleep), sleep-disordered breathing (three items, e.g. snores loudly), and daytime sleepiness (eight items, e.g. seems tired).

Parents were required to rate the frequency of certain sleep-related behaviors that occurred in the previous week. A response of “usually” (given a score of 3) indicated that the behavior had occurred between five and seven times, “sometimes” (scored 2) meant it had happened two to four times, and “rarely” (scored 1) meant that the behavior was observed once at most. The reliability and validity of the instrument had been verified in a sample of Chinese primary school students [30]. The Cronbach’ s alpha coefficient in the current sample was 0.75, which was similar to other studies [22, 23]. Parents were required to report the time their children fell asleep and woke up on weekdays and weekends. Sleep length was calculated by the investigators.

#### Bedtime activities

##### Diet behaviors

Children were asked to rate the frequency of their consumption of snacks, soft drinks, tea, and coffee before bedtime in a typical week on a five-point Likert scale ranging from 0 (never) to 4 (seven times a week).

##### Screen time

Children were asked to rate how frequently they used a smart phone or computer (including an or any electronic music player, tablet, or other device) or watched TV before bedtime in a typical week using a five-point Likert scale ranging from 0 (never) to 4 (seven times a week).

### Anxiety/depression symptoms

Parents filled out the subscale of the Achenbach Child Behavior Checklist (CBCL, the version fit for children ages 6–18 years old) to evaluate children’s anxiety/depression symptoms. The subscale of anxiety/depression included 17 items, for example, fears he / she might think or do something bad; Feels or complains that no one loves him / her. Parents were required to rate the frequency of each anxiety/depression symptom over the past 6 months on a three-point Likert scale (0 = not true, 1 = sometimes true, and 2 = often true). The reliability and validity of the instrument had been verified among Chinese children and teenagers [31]. The Cronbach’ s alpha coefficient in the current sample was 0.87.

### Data analysis

**Fig. 1:**
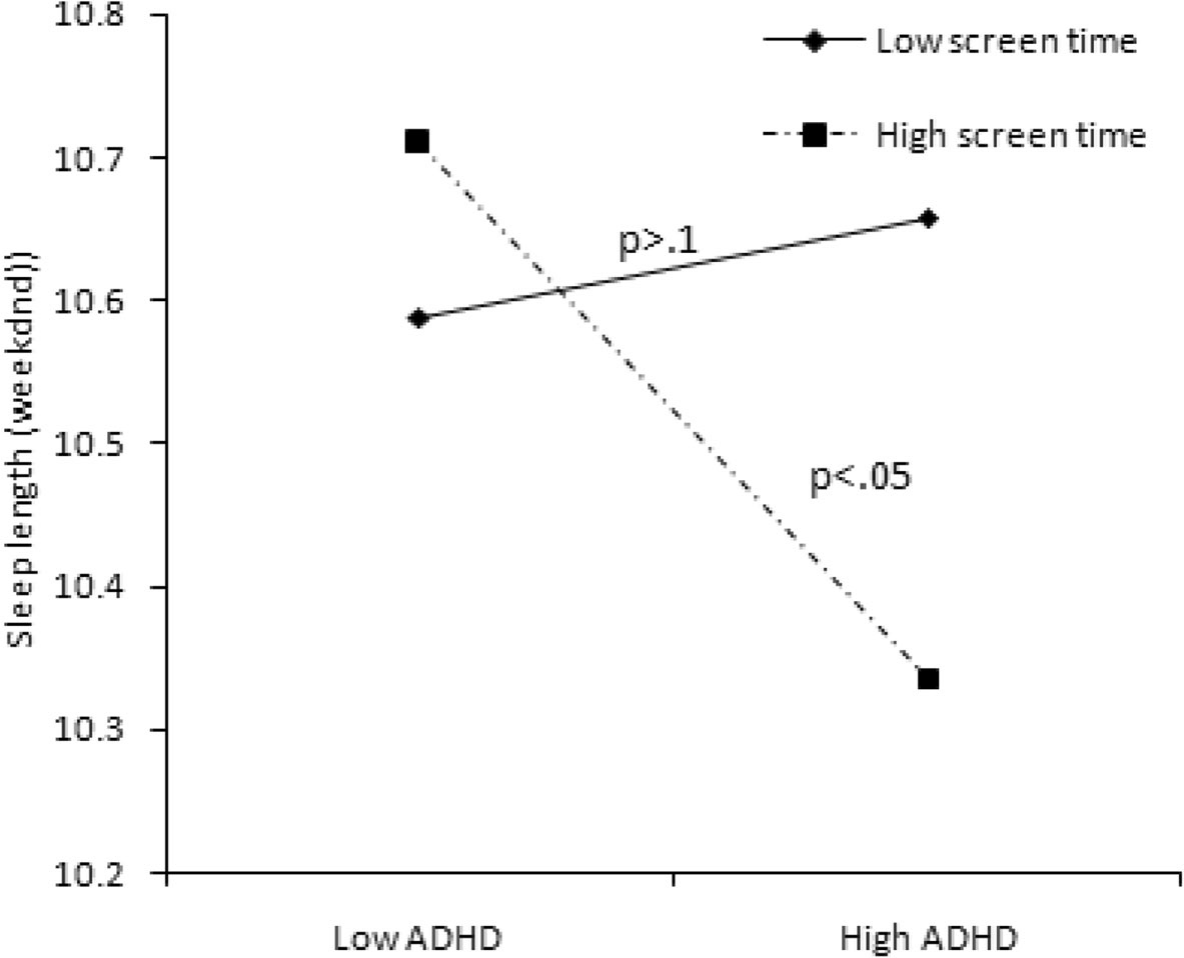
Effect of screen time × ADHD symptoms interaction on sleep length

**Fig. 2:**
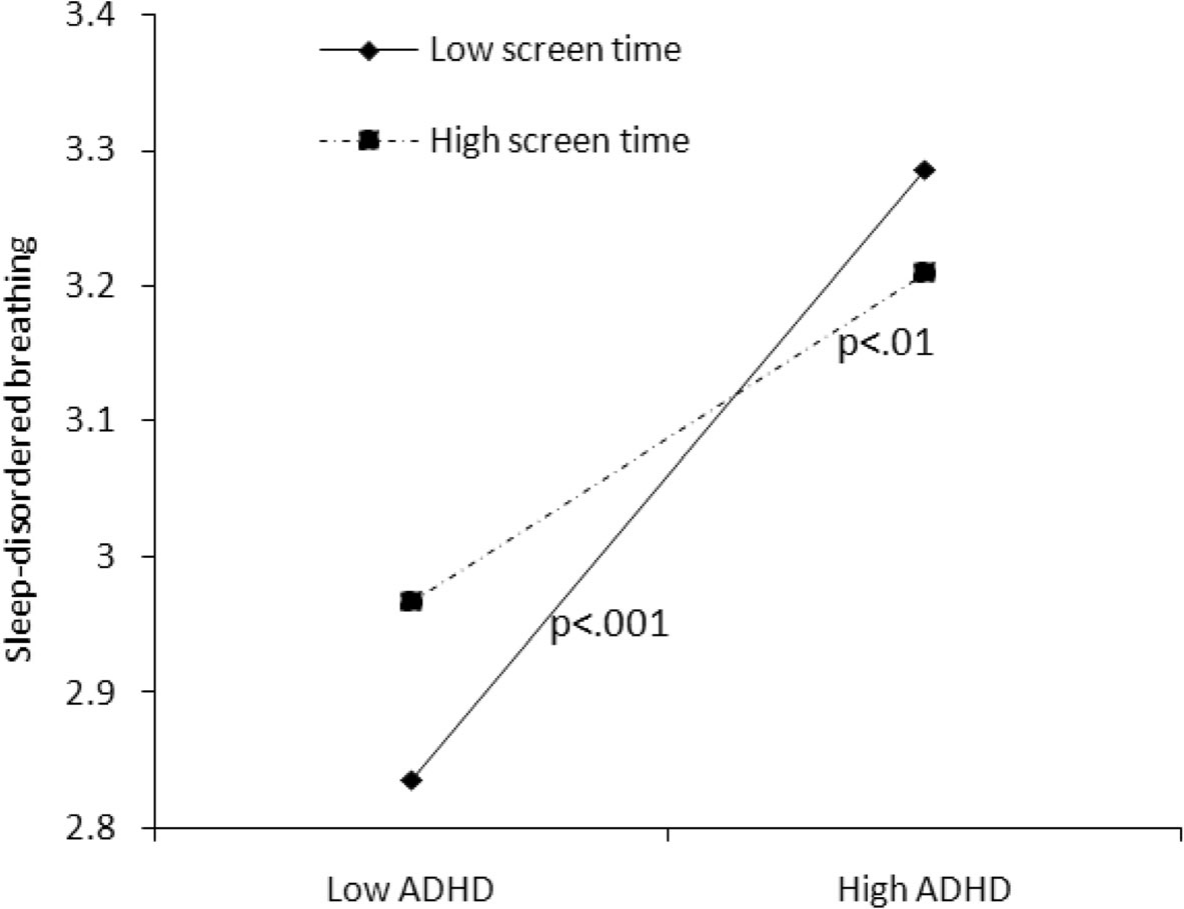
Effect of screen time × ADHD symptoms interaction on sleep-disordered breathing

**Fig. 3:**
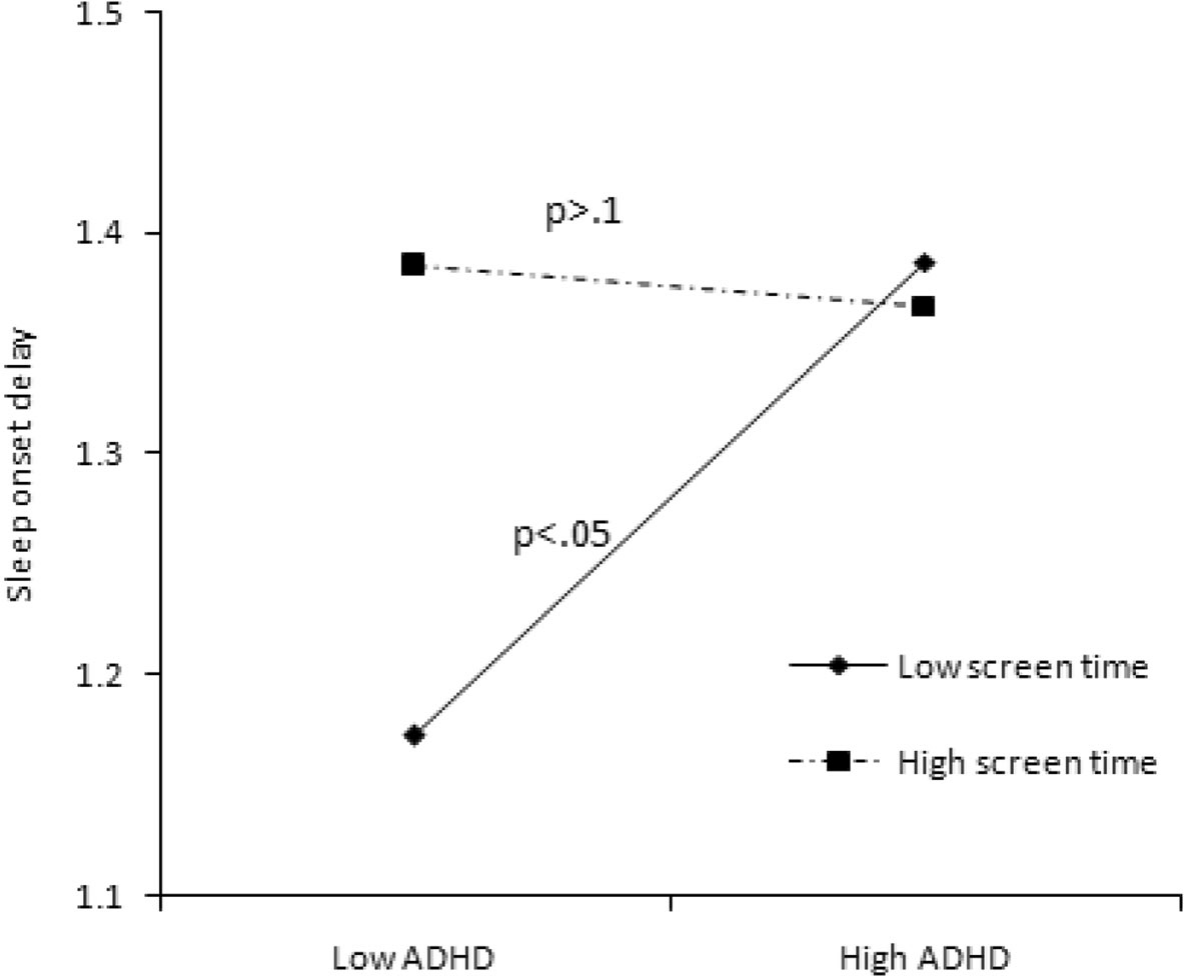
Effect of screen time × ADHD symptoms interaction on sleep onset delay

**Fig. 4:**
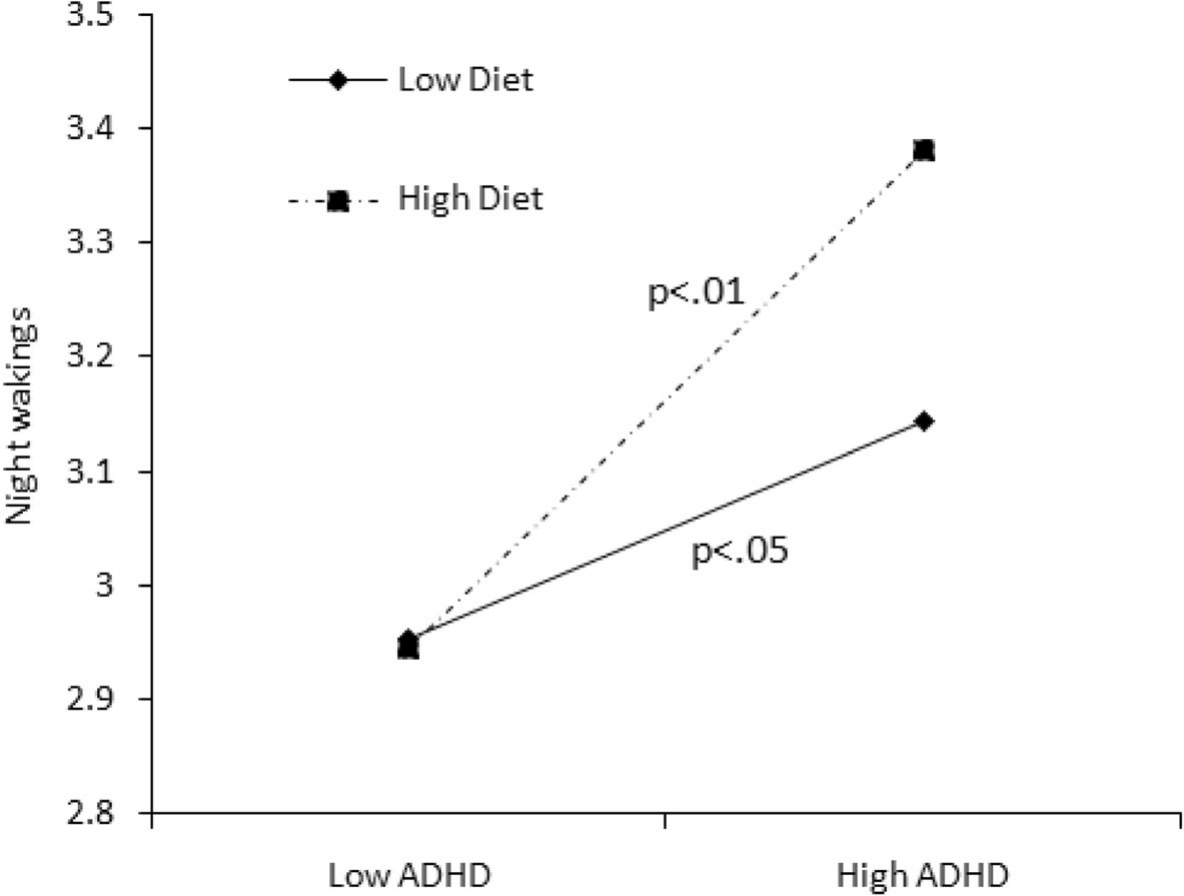
Effect of diet × ADHD symptoms interaction on night wakings

**Fig. 5:**
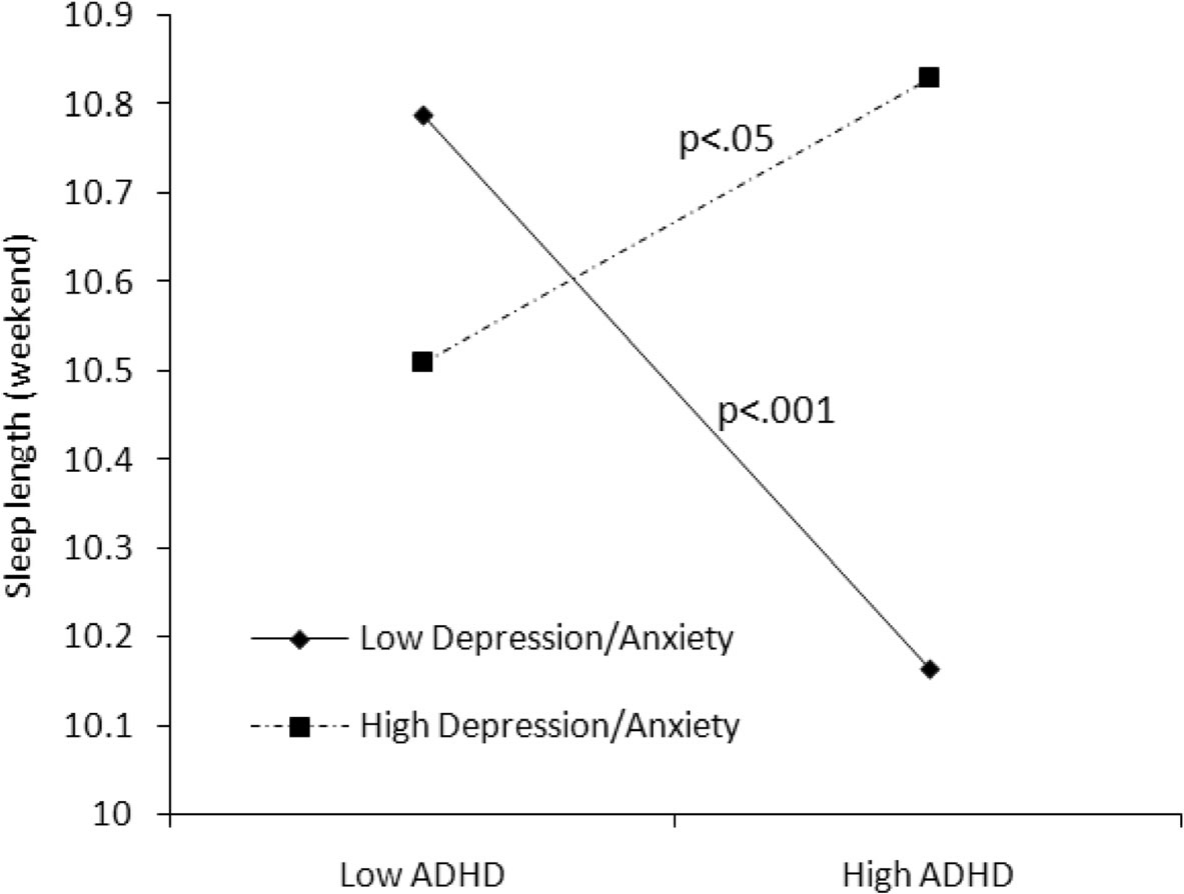
Effect of anxiety/depression × ADHD symptoms interaction on sleep length

**Fig. 6:**
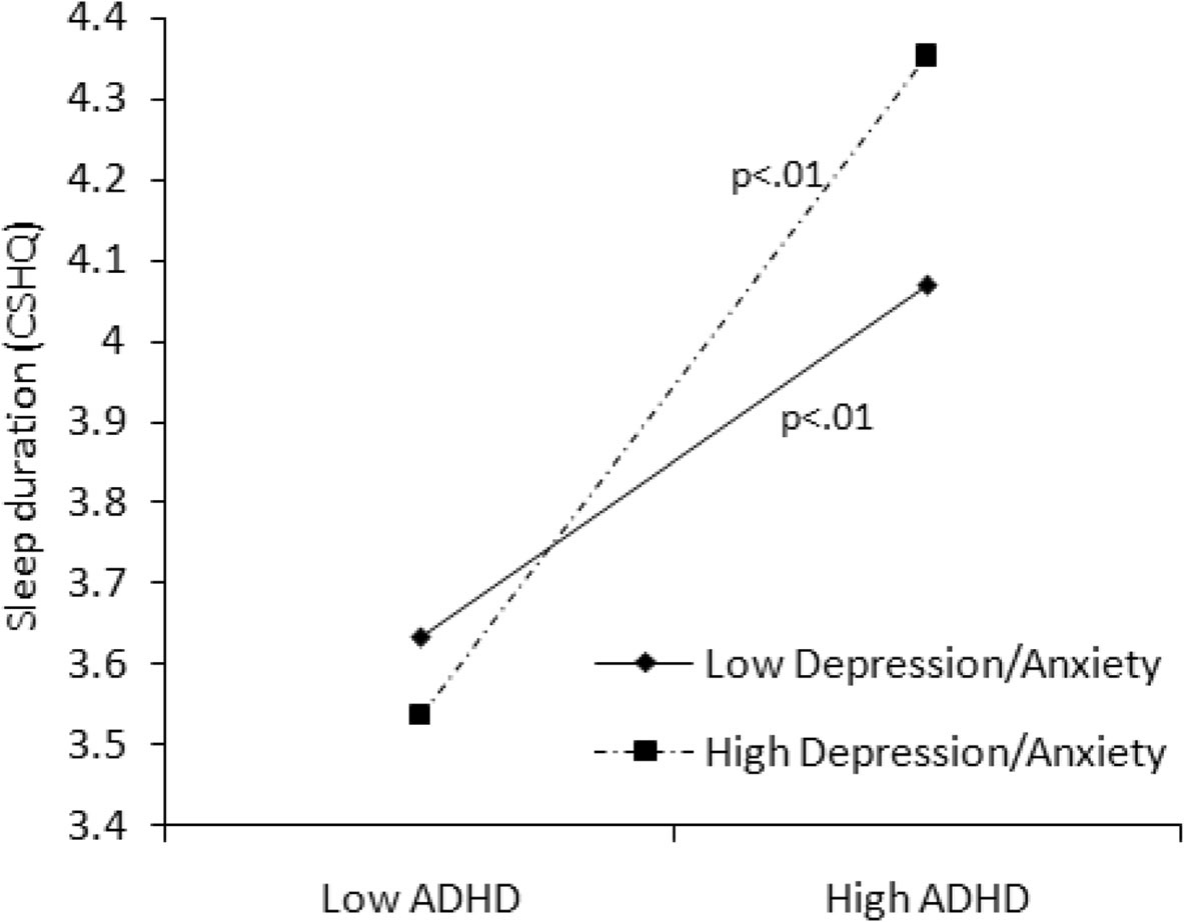
Effect of anxiety/depression × ADHD symptoms interaction on sleep duration problems

We conducted statistical analyses with SPSS version 20.0. The cutoff point of 26 was used to define children with or without ADHD. A chi-squared test was used to examine the differences in categorical variables of demographic information between the ADHD symptomatic and non-symptomatic groups, while a t-test was used for continuous variables (i.e., age). The Wilcoxon rank-sum test was used to clarify the differences in sleep length, sleep problems, bedtime activities, and anxiety/depression between two ADHD groups. Additionally, we used hierarchical regression analyses to examine the predictors of sleep length and sleep problems and the interactive effect of moderators. Both moderators and independent variables were centralized by a Z-score to avoid potential multicollinearity among the variables in the regression equation [32]. The moderating effects of bedtime screen time and diet and anxiety/depression are shown in Figs. 1, 2, 3, 4, 5 and 6.

## Results

Table 1 presents the demographics of the participants. Of the 934 children, 82 (10.3%) belonged to the ADHD symptomatic group, and more boys (14.5%) were symptomatic than girls (6.1%; *χ*^2^ = 14.456, *P* < 0.01). The proportion of ADHD children did not differ by grades (*χ*^2^ = 2.187, *P* > 0.1). The mean age of the ADHD symptomatic group was 10.3 years, slightly younger than the ADHD non-symptomatic group (10.6; *t* = 2.691, *P* < 0.01). No significant differences in parents’ education level (father: *χ*^2^ = 3.172, *P* > 0.1; mother: *χ*^2^ = 1.634, *P* > 0.1) and annual household income (*χ*^2^ = 2.187, *P* > 0.1) were found between the ADHD symptomatic and non-symptomatic groups, which means the proportion of non-symptomatic and symptomatic children was similar across parents’ education levels and across household incomes.

**Table 1:** Characteristics of study populations with and without ADHD symptoms

	Total % (n)	ADHD		*χ* ^2^
		Non-Symptomatic % (n)	Symptomatic % (n)	
Gender				
Male	53.8 (501)	85.5 (428)	14.5 (73)	14.456***
Female	46.2 (430)	93.9 (404)	6.1 (26)	
Grade				
Three	36.6 (342)	87.3 (299)	12.7 (43)	2.187
Four	30.0 (280)	90.6 (253)	9.4 (27)	
Five	33.4 (312)	90.8 (283)	9.2 (29)	
Father’s education level				
Primary school and below	3.7 (28)	89.3 (25)	10.7 (3)	3.172
Junior high school	37.4 (286)	88.1 (252)	11.9 (34)	
Senior high school	26.8 (205)	89.8 (184)	10.2 (21)	
College	29.5 (226)	92.5 (209)	7.5 (17)	
Graduate	2.6 (20)	85.0 (17)	15.0 (3)	
Mother’s education level				
Primary school and below	11.9 (91)	87.9 (80)	12.1 (11)	1.634
Junior high school	35.8 (273)	88.6 (242)	11.4 (31)	
Senior high school	24.0 (183)	90.2 (165)	9.8 (18)	
College	27.7 (211)	91.0 (192)	9.0 (19)	
Graduate	0.7 (5)	100 (5)	0	
Annual household income (USD)				
≤ 3000	6.9 (50)	88.0 (44)	12.0 (6)	1.080
3000–6000	11.8 (86)	88.4 (76)	11.6 (10)	
6000–9000	19.5 (142)	88.7 (126)	11.3 (16)	
9000–12,000	14.4 (105)	91.4 (96)	8.6 (9)	
12,000–15,000	17.5 (127)	89.8 (114)	10.2 (13)	
≥ 15,000	29.8 (217)	90.8 (197)	9.2 (20)	
Total	100.0 (934)	91.2 (852)	8.8 (82)	

***P < 0.01

We found that children’s average sleep time was 9.3 h on weekdays, significantly less than the average 10.1 h on weekends (*t* = − 18.152, *P* < 0.001; Table 2). However, there was no difference in sleep hours between the ADHD symptomatic group and non-symptomatic group. The ADHD symptomatic group had more sleep problems in general. Compared with the non-symptomatic group, the symptomatic group had more bedtime resistance problems (*U* = 14,984.5, *P* < 0.001), sleep onset delay (*U* = 24,284.5, *P* < 0.05), sleep duration problems (*U* = 17,729.0, *P* < 0.001), sleep anxiety problems (*U* = 20,815.0, *P* < 0.05), night waking problems(*U* = 20,552.5, *P* < 0.0001), parasomnias problems (*U* = 17,845.0, *P* < 0.0001), sleep-disordered breath problems (*U* = 20,407.0, *P* < 0.0001), and daytime sleepiness problems (*U* = 17,546.0, *P* < 0.0001) (Table 2). In addition, children in the ADHD symptomatic group had more screen time (*U* = 22,595.0, *P* < 0.05) and consumed more snacks, soft drinks, tea, and coffee before bedtime than children without ADHD symptoms (*U* = 22,705.0, *P* = 0.071). Furthermore, children in the ADHD symptomatic group had more anxiety/depression symptoms than those in the non-symptomatic group (*U* = 13,516.0, *P* < 0.001).

**Table 2:** The differences in sleep status, bedtime activities, and anxiety/depression between the two groups

	ADHD		*U*
	Non-Symptomatic $\overline{\mathrm{x}}$ (*SD*)	Symptomatic $\overline{\mathrm{x}}$ (*SD*)	
Sleep length			
Weekdays	9.3 (0.6)	9.3 (0.7)	25,424.0
Weekends	10.1 (0.9)	10.3 (2.8)	26,846.0
Sleep problems (total score)	46.3 (6.7)	52.9 (6.9)	9237.0***
Bedtime resistance	8.2 (2.2)	9.6 (2.6)	14,984.5***
Sleep onset delay	1.4 (0.6)	1.6 (0.7)	24,284.5*
Sleep duration	4.4 (1.4)	5.3 (1.5)	17,729.0***
Sleep anxiety	5.4 (1.8)	6.1 (1.9)	20,815.0*
Night waking	3.5 (0.8)	4.1 (1.2)	20,552.5***
Parasomnias	8.4 (1.4)	9.3 (1.9)	17,845.0***
Sleep-disordered breath	3.3 (0.6)	3.8 (1.2)	20,407.0***
Daytime sleepiness	11.8 (2.8)	13.1 (2.7)	17,546.0***
Bedtime activities			
Screen activities	5.3 (2.2)	6.0 (2.9)	22,595.0*
Diet	4.7 (1.9)	5.4 (2.9)	22,705.0^+^
Anxiety/depression	2.7 (2.9)	5.9 (4.1)	13,516.0***

^+^*P* < 0.1, *P < 0.05, ***P < 0.0001

The results of correlation analysis were presented in Table 3. After controlling for children’s gender and age and parents’ education and annual household income, ADHD symptoms were significantly correlated with sleep problems (*r* = .48, *p* < .001), sleep length on weekday (*r* = −.11, *p* < .05), diet behavior (*r* = .14, *p* < .01), and anxiety/depression problems (*r* = .24, p < .001). It was found that sleep problems were associated with diet behavior (*r* = .12, p < .01), and anxiety/depression problems (*r* = .69, p < .001), and only marginally associated with sleep length on weekday(*r* = −.10, *p* = .02) and screen activities (*r* = .08, *p* = .08).

**Table 3:** Correlations among the study variables

Main variables	1	2	3	4	5	6
1. ADHD symptoms	1					
2. Sleep problems	.48***					
3. Sleep length (weekday)	−.11*	−.10^+^				
4. Sleep length (weekend)	.01	.06	.21***			
5. Bedtime activities (screen)	.05	.08^+^	.02	−.02		
6. Bedtime activities (diet)	.14**	.12*	.04	−.02	.55***	
7. Anxiety/depression	.24***	.69***	−.08^+^	0	.05	.08^+^

^+^*P* < 0.1, *P < 0.05, **P < 0.001, ***P < 0.0001

We used hierarchical linear regression analysis to examine the predictors of ADHD, bedtime activities, anxiety/depression, and each sleep problem. The main findings are shown in Table 4. In Model 2, after controlling for children’s gender and age and parents’ education and annual household income, ADHD symptoms significantly predicted children’s sleep length on weekdays (*B* = − 0.107, *P* < 0.001), total sleep problems (*B* = 2.792, *P* < 0.001), bedtime resistance problems (*B* = 0.625, *P* < 0.001), sleep duration problems (*B* = 0.325, *P* < 0.001), sleep anxiety problems (*B* = 0.314, P < 0.001), night waking problems(*B* = 0.164, *P* < 0.001), parasomnias problems (*B* = 0.361, *P* < 0.001), sleep-disordered breathing problems (*B* = 0.170, *P* < 0.001), and daytime sleepiness problems(*B* = 0.779, *P* < 0.001). Anxiety/depression was another important factor associated with weekend sleep time (*B* = 0.195, *P* < 0.01) and total sleep problems (*B* = 1.413, *P* < 0.001).

**Table 4:** Hierarchical linear regression analysis

	Model 1^a^	Model 2^b^	Model 3^c^
	Beta	Beta	Beta
Sleep length(weekend)			
Child’s gender	.134	.088	.086
Child’s age	−.074	−.079	−.065
Father’s education	−.118	−.119	−.116
Mother’s education	−.110	−.107	−.121
Annual household income	−.012	−.007	−.007
ADHD		−.080	−.076
Screen behaviors in bedtime (Screen)		−.038	−.050
Diet behaviors in bedtime		.003	.044
Anxiety/Depression		.195**	.097
Screen × ADHD			−.111*
Diet × ADHD			−.027
Anxiety/Depression × ADHD			.236***
*R*^*2*^*(F)*	.029 (3.7**)	.047 (3.4***)	.094 (5.3***)
Sleep problems (total)			
Child’s gender	−.029	1.260	1.267*
Child’s age	−.358	.096	.102
Father’s education	−.127	.108	.113
Mother’s education	−.902*	−.530	−.531
Annual household income	−.301	−.354*	−.353*
ADHD		2.792***	2.811***
Screen behaviors in bedtime		.504	.545^+^
Diet behaviors in bedtime		.087	.037
Anxiety/Depression		1.413***	1.437***
Screen × ADHD			−.272
Diet × ADHD			.165
Anxiety/Depression × ADHD			−.060
*R*^*2*^*(F)*	.034(3.649***)	.292(23.820***)	.293(17.874***)
Sleep onset delay			
Child’s gender	.029	.057	.062
Child’s age	.021	.026	.031
Father’s education	.061	−.060	−.061
Mother’s education	−.088*	−.076*	−.075*
Annual household income	.009	.010	.010
ADHD		.039	.049
Screen behaviors in bedtime		.038	.048
Diet behaviors in bedtime		.001	.005
Anxiety/Depression		.025	−.025
Screen × ADHD			.058*
Diet × ADHD			.0
Anxiety/Depression × ADHD			−.002
*R*^*2*^*(F)*	.044(5.6***)	.054(3.9***)	.064(3.5***)
Sleep duration			
Child’s gender	.050	.192	.188
Child’s age	.051	.097^+^	.100^+^
Father’s education	−.054	−.026	−.025
Mother’s education	−.157^+^	−.140^+^	−.145^+^
Annual household income	−.083*	−.086*	−.085*
ADHD		.325***	.313***
Screen behaviors in bedtime		.020	.002
Diet behaviors in bedtime		.031	.046
Anxiety/Depression		.087	.046
Screen × ADHD			.033
Diet × ADHD			−.021
Anxiety/Depression × ADHD			.095^+^
*R*^*2*^*(F)*	.041 (5.0***)	.102 (7.4***)	.107 (5.8***)
Night wakings			
Child’s gender	.039	.108	.103
Child’s age	.045	.075*	.077*
Father’s education	−.043	−.027	−.029
Mother’s education	−.160**	−.152**	−.152**
Annual household income	.018	.019	.021
ADHD		.164***	.157***
Screen behaviors in bedtime		−.025	−.018
Diet behaviors in bedtime		.076^+^	.057
Anxiety/Depression		.065	.050
Screen × ADHD			−.043
Diet × ADHD			.061^+^
Anxiety/Depression × ADHD			.049
*R*^*2*^*(F)*	.046 (5.7***)	.098 (7.2***)	.107 (5.9***)
Sleep-disordered breathing			
Child’s gender	−.133*	−.061	−.061
Child’s age	.010	.035	.036
Father’s education	−.021	−.009	−.010
Mother’s education	−.018	−.011	−.009
Annual household income	.010	.009	.010
ADHD		.170***	.173***
Screen behaviors in bedtime		.003	.014
Diet behaviors in bedtime		.025	.014
Anxiety/Depression		−.009	−.008
Screen × ADHD			−.052^+^
Diet × ADHD			.036
Anxiety/Depression × ADHD			.006
*R*^*2*^*(F)*	.012 (1.5)	.067 (4.8***)	.074 (3.9***)

^+^*p* < .1, *p < .05, **p < .001, ***p < .01

^a^Model 1: Gender, age, father’s education, mother’s education, Annual household income. These variables were adjusted in model 2–3;

^b^Model 2: Variables of ADHD, Screen behavior (Screen), Diet before sleep (Diet), Anxiety/Depression were added;

^c^Model 3: Interactive terms of Screen × ADHD, Diet × ADHD, Anxiety/Depression× ADHD were added

In Model 3, when the interaction effect with ADHD was taken into consideration, bedtime activities more strongly predicted sleep length and sleep problems (see Table 4). We found that the interaction between screen time and ADHD predicted sleep duration on weekends (*B* = − 0.111, *P* < 0.05), sleep onset delay (*B* = − 0.058, *P* < 0.05), and sleep-disordered breathing (*B* = − 0.052, *P* = 0.057). These interactive effects are presented in Figs. 1–4. For children with low screen time before bedtime, sleep length decreased with increasing ADHD symptoms (Fig. 1). For children with low as well as with high screen time before bedtime, sleep disorder breathing problems increased with increasing ADHD symptoms (Fig. 2). For children with low screen time before bedtime, sleep onset delay exacerbated with increasing ADHD symptoms (Fig. 3). Additionally, eating before bedtime was associated with longer sleep length on weekdays (*B* = 0.070, *P* < 0.05) and more night waking problems (*B* = 0.076, *P* < 0.1). Furthermore, eating before bedtime played a moderating role in the relationship between ADHD and night waking (*B* = 0.061, *P* < 0.1). For children with low as well as with high screen time before bedtime, night waking problems increased with increasing ADHD symptoms (Fig. 4).

Taking emotional problems into account, anxiety/depression exacerbated total sleep problems (*B* = 1.413, *P* < 0.001), particularly sleep anxiety (*B* = 0.285, *P* < 0.001), parasomnias (*B* = 0.305, *P* < 0.001), and daytime sleepiness (*B* = 0.488 *P* < 0.001). In Model 2, we found that more anxiety/depression symptoms predicted longer sleep duration on weekends (*B* = 0.195, *P* < 0.01). Furthermore, in Model 3, the interaction of anxiety/depression and ADHD predicted longer sleep periods on weekends (*B* = 0.236, *P* < 0.001) as well as sleep duration problems (*B* = 0.095, *P* = 0.08). The interaction effects illustrated in Fig. 5 show that for children with low Depression/Anxiety scores, sleep duration on weekends decreased with increasing ADHD symptoms while for children with high Depression/Anxiety scores, sleep duration rather increased with increasing ADHD symptoms.

For children with low as well as with high Depression/Anxiety scores, sleep duration problems increased with increasing ADHD symptoms (Fig. 6). Additionally, the mother’s educational attainment independently contributed to children’s sleep problems in general (*B* = − 0.902, *P* < 0.05) and was related to sleep onset delay (*B* = − 0.088, *P* < 0.05), sleep duration problems (*B* = − 0.157, *P* < 0.1), and night waking (*B* = − 0.160, *P* < 0.05), as well as parasomnias (*B* = − 0.231, *P* < 0.05).

## Discussion

Children with ADHD often experience sleep problems [33], and this association was supported by the present study. We found that children with more ADHD symptoms had more bedtime resistance problems, sleep onset delay, sleep duration problems, sleep anxiety, night waking problems, parasomnias, sleep-disordered breath, and daytime sleepiness problems. Other findings of the present study offered new evidence for interpreting the potential mechanisms. For example, we found that children with ADHD symptoms had more unhealthy bedtime activities, including more screen time and eating. These behaviors associated with sleep problems independently in addition to moderating the relationship between ADHD symptoms and sleep problems.

Specifically, children with more ADHD symptoms and screen time before bedtime tended to have shorter sleep times on weekends. A previous study also suggested that high exposure to games and TV decreased sleep hours among boys diagnosed with ADHD [17]. This may be because parents allow their children to use electronics before bedtime for longer periods on weekends than on weekdays, so as to reduce unexpected behaviors for children with ADHD. Additionally, we found bedtime screen activities to be a strong moderating factor for the relationship between ADHD symptoms and sleep-disordered breathing problems. Children with high ADHD symptoms showed more sleep-disordered breathing problems than those with low ADHD symptoms; moreover, both low screen time and high screen time significantly strengthened this association. This suggests that even a short period of exposure to screen activities before bedtime may make children with ADHD symptoms more hyperactive and impulsive [16, 34], which may lead to more breathing problems and snoring [35].

With regard to the problem of sleep onset delay, neither ADHD symptoms nor screen time associated with sleep onset delay independently; however, the interaction of the two factors significantly affected sleep onset delay. Regardless of ADHD symptoms, children with high screen time always experienced longer sleep onset delay, while the children with low screen time experienced more sleep onset delay with increasing ADHD symptoms. This suggests that children with ADHD symptoms should avoid screen activities before bedtime, because even a short screen time may excite them and make it hard for them to fall asleep. This explanation is supported by the fact that violent or age-inappropriate content, the fast pace of entertainment media, and higher overall screen time can lead to ADHD-related behaviors (e.g., intense arousal, poor self-control) [13].

Moreover, we found that bedtime diet moderated the relationship between ADHD symptoms and sleep problems. More bedtime eating behaviors were associated with more night waking problems with increasing ADHD symptoms. As of now, the mechanism of the relationship between bedtime diet and ADHD symptoms is unclear. Children with ADHD often have eating disorders, which may increase the risk of night eating behaviors. Our previous study also demonstrated that children with ADHD symptoms showed more frequent bedtime eating behaviors than non-ADHD children [19]. Another study showed that young adults with night eating syndrome were significantly more likely to have histories of ADHD, depression, and eating disorders [36]. These studies showed that hypersensitivity to food may play an etiologic role in sleep complaints in children with ADHD and suggest a relationship between diet and sleep problems in children with ADHD. However, previous studies of night eating syndrome have been conducted in clinical samples. Our study findings add to the literature suggesting that bedtime eating may associate with sleep problems in general school-age children.

An interesting finding of the current study was that emotional problems (i.e., anxiety/depression) played a moderating role in the relationship between ADHD symptoms and sleep problems. Children with more ADHD symptoms and high levels of anxiety/depression had longer sleep durations on weekends, but poorer sleep quality (wake up more during the night). The finding is consistent with a recent study, which found that that children combined ADHD and anxiety/depression had shorter sleep durations and more sleep problems than their counterparts [37]. It also suggests that children with ADHD may have different sleep patterns on weekdays and weekends. Especially for school-age children in China, the strict school schedule on weekdays is a great challenge for children with mental health problems, and to compensate, they sleep longer on weekends. As shown in the present study, children with ADHD and emotional problems experienced more sleep problems, so they were likely tired on weekdays and slept longer on weekends to compensate. Furthermore, the parent-reported sleep times for children may be biased, because the time the child goes to bed may not equal the time the child falls asleep, especially for children with emotional problems. It has been suggested that anxiety/depression symptoms may increase nighttime fears related to personal safety, separation, loss, pressures, and so on, leading these children to stay awake in bed or even lose sleep at night [23].

Additionally, a mother’s higher level of education appears to be a protective factor for some sleep problems, including sleep onset delay, sleep duration, night waking, and parasomnias. Some previous studies showed that parental education and SES were associated with sleep hours and sleep problems. For example, one study found that higher parental education was associated with longer sleep time in their children [38]. Children in low-SES families have more sleep problems than children in high-SES families [39, 40]. The mother is generally the primary caregiver in both Western countries and Asian countries [41] and therefore tends to have a greater impact on the child. Mothers with higher education know more about healthy sleep habit and are more likely to provide their children regular bedtimes and bedtime routines [42]. One evidence-based survey reported that parental control of night activities could considerably improve children’s sleep quality [43].

### Limitations

Some limitations of this study should be mentioned. First, the study design was a cross-sectional design, limiting our ability to draw causal inferences. For example, it is impossible to clarify whether media use causes sleep onset difficulties or whether children with ADHD who have trouble falling asleep turn to electronics as a way to cope with their sleep onset problems. Second, the sleep hours and sleep problems were assessed by parent-reported rating scales rather than measured by devices, so the data may have been imprecise. Child report of sleep problems was not collected - this is particularly important for aspects of sleep that parents may not observe such as night wakings and parasomnias. Third, this study used a questionnaire and not an interview-based assessment to assess ADHD symptoms. Forth, the data on medication was not collected, so the role of medication in these relationships is unknown. Therefore, more research is needed to determine the causal effects of bedtime activities on sleep problems in children with ADHD.

## Conclusions

Bedtime activities and emotional problems had important moderating effects on the relationship between ADHD and sleep problems. Bedtime management—including consistent parent-set bedtimes, a clear structure surrounding house rules, and bedtime behavior management—could reduce sleep problems for children with ADHD. Moreover, paying attention to children’s emotional status and strengthening their emotion management could potentially improve sleep quality as well.

## Abbreviations

ADHD: Attention Deficit Hyperactivity Disorder;
ADHDRS-IV: ADHD Rating scale-IV;
CBCL: Achenbach Child Behavior Checklist
CSHQ: Children’s Sleep Habits Questionnaire
DSM-IV: Diagnostic and Statistical Manual of Mental Disorders-IV;
SES: Social-economic Status
